# Double trouble: how co- and superinfections shape viral dynamics and host responses

**DOI:** 10.1128/jvi.02055-25

**Published:** 2026-06-23

**Authors:** Marta Bermejo-Jambrina, Mindaugas Paužuolis, Janine Kimpel, Gisa Gerold

**Affiliations:** 1 Department of Hygiene, Microbiology and Virology, Institute of Virology, Medical University of Innsbruck 27280, Innsbruck, Austria; Indiana University Bloomington, Bloomington, Indiana, USA

**Keywords:** superinfection, coinfection, virus-virus interaction, superinfection exlcusion (SIE), infection, human immunodeficiency virus, coronavirus, host immune response, clinical outcome

## Abstract

Concurrent infection of humans by multiple distinct viruses is a common biological phenomenon with important consequences for virus-host interactions. While coinfection refers to the simultaneous infection of an individual or cell with two or more viruses, superinfection denotes the establishment of a secondary infection following a primary one, in which the initial infection influences the susceptibility, replication, or outcome of the secondary infection. Virus-virus interactions can be antagonistic, facilitative, dependent/assisted, or neutral. Antagonistic interactions include superinfection exclusion, whereby a primary virus prevents or restricts secondary infection of the same cell, securing access to host resources and stabilizing replication while limiting genetic and viral diversity. In contrast, facilitative viral interactions arise when one virus disrupts tissue barriers, suppresses immune responses, or remodels cellular pathways, thereby increasing susceptibility to additional viral infections. These interactions intersect with fundamental viral strategies, including the generation of genetically diverse RNA virus quasispecies that promote rapid adaptation and the deployment of DNA virus immunoevasins that interfere with antigen presentation and host immune recognition. While each of these processes has been extensively studied individually, their combined impact during coinfection remains poorly defined. In this review, we synthesize current knowledge on virus-virus interactions, viral diversity, and immune evasion in the context of multi-viral infections. We focus on how interactions at cellular and tissue levels shape infection outcomes, influence viral evolution, and contribute to virus-host coevolution. Finally, we also highlight key gaps in our understanding of viral interference and cooperation and discuss emerging approaches needed to define the spatiotemporal dynamics of coinfection *in vivo*.

## GENERAL FEATURES OF VIRAL COINFECTION AND SUPERINFECTIONS

### Viral coinfection and superinfection as common biological phenomena

Infection by multiple viruses is common in nature and can occur either concurrently or sequentially within the same host or cell. While coinfection refers to the simultaneous infection of an individual or cell with two or more viruses, superinfection denotes the establishment of a secondary infection following a primary one, in a context where the primary infection influences susceptibility, replication, or outcome of the secondary infection (1). These virus-virus interactions within the host can occur between unrelated viruses or strains of the same virus. Accordingly, such events are frequent in both acute and chronic infections and can result in diverse outcomes ranging from inhibition to enhancement of viral replication (2, 3). Clinically, viral coinfections are increasingly reported, with global medical importance, including human immunodeficiency virus (HIV), hepatitis viruses, and dengue virus (DENV). In the respiratory tract particularly, concurrent or sequential infections with respiratory pathogens, including influenza viruses, rhinovirus (RV), and human coronaviruses, may occur in a single host due to the global co-circulation of these viruses, especially during winter epidemics (4–6). Beyond shared routes of transmission, coinfections with pathogens transmitted via different routes (e.g., respiratory and insect-borne) are increasingly common in areas with a high burden of both diseases (7–10).

### Epidemiology and immunological context of viral coinfections

Consistent with this view, heterologous immunity recognizes that humans (and animals) live in constant interaction with multiple pathogens. Although most immunological research traditionally focuses on single-pathogen infections, viral coinfections, whether simultaneous or sequential, are the rule rather than the exception in natural settings. Epidemiological evidence from respiratory, enteric, and vector-borne viruses shows that up to ~10%–20% of viral infections involve more than one virus (11, 12). However, data remain scarce, especially for unrelated coinfections; the global burden of acute viral coinfections on human health is still largely undetermined (3, 13).

### Outcomes of viral coinfection: antagonism, synergy, dependence, and neutrality

Coinfection outcomes range from mutual inhibition (antagonism) to synergistic enhancement of replication, or neutral coexistence without measurable interaction (13, 14). The type of interaction depends on viral tropism, timing, host immune status, dose, and tissue localization. Such immune alterations translate directly into epidemiological and clinical phenomena, as seen in respiratory virus circulation patterns and vaccine responses.

### Multiscale mechanisms of virus-virus interaction

At the cellular level, when different viruses infect the same cell, interactions may range from direct competition for host resources to modulation of replication. Several patterns of virus-virus interactions have been described: interference, synergy, noninterference, dependence/assistance, and host-parasite-like relationships. Noninterference occurs when coinfecting viruses replicate independently without affecting each other, while host-parasite-like relationships describe cases where one virus exploits components or replication products of another virus to facilitate its own replication. Viral coinfection and superinfection are not rare events; instead, virus-virus interactions shape viral replication dynamics and host immune responses at the cellular level and provide a mechanistic foundation for understanding complex outcomes of infection with important clinical consequences (15, 16).

## DETERMINANTS AND WITHIN-HOST MECHANISMS OF VIRUS-VIRUS INTERACTION

The outcome of viral coinfection is determined by within-host processes that unfold at the cellular and local tissue level. Viruses may interact within the same infected cell, or indirectly through paracrine signaling, innate immune activation, altered receptor availability, and changes in the local tissue environment. These encounters can produce several distinct outcomes, including interference, superinfection exclusion (SIE), synergy or facilitation, noninterference, dependence or assistance, and complementation. Which outcome predominates depends strongly on viral tropism, timing and sequence of infection, multiplicity of infection (MOI), or viral burden, the compatibility of replication niches, and the host immune state.

### Antagonistic interactions: interference and superinfection exclusion

Antagonistic interactions occur when one virus directly or indirectly reduces the entry, replication, or spread of another virus. Among these interaction classes, interference is the most frequent and can be mediated by viral proteins, competition for host factors, receptor modulation, innate immune activation, or exclusion from replication complexes. Hepatotropic viruses provide a clear example of this antagonism. In hepatitis C and hepatitis B (HCV/HBV) coinfection, the HCV core protein directly represses HBV enhancer activity and polymerase expression, thereby suppressing HBV replication (17–22). Clinically, viral dominance is context-dependent and can shift with viral load; that is, mixed HCV/HBV infections, antiviral therapy such as interferon (IFN) therapy, and intra-host variants can determine which virus prevails (18, 22).

Interference can also be mediated by host innate immunity. A protective IFN-mediated innate immune response can persist even after, in sequential infection, the first virus was eliminated. For example, for respiratory viruses, dual coinfection or sequential infection of influenza A virus (IAV) H1N1 or respiratory syncytial virus A (RSV-A) with RV-A16 leads to RV-A16 inhibition due to its high sensitivity to type I and type III IFN responses (23, 24). Non-IFN-mediated, “intrinsic” interference also occurs, for example, when Newcastle disease virus creates a refractory state through viral-encoded proteins, or Semliki Forest virus (SFV) blocks attachment, entry, and uncoating of a secondary virus (3). Additional mediators include defective interfering particles, RNA interference, trans-acting viral proteins, and nonspecific dsRNA.

SIE is a specific form of interference and the most pervasive cellular strategies shaping viral ecology. It describes a state in which an established infection prevents or restricts subsequent entry or replication of a second virus, thus its infection of the same cell by another virus of the same or a closely related species. SIE can operate at multiple stages of the viral life cycle, including receptor binding, membrane fusion, translation, or replication (25–33).

#### Entry, fusion, and uncoating blocks

At the cell surface, SIE can be mediated by receptor downregulation, altered entry factor availability, or post-attachment blocks to membrane fusion and uncoating. In HIV-1, SIE is achieved through coordinated modulation of surface receptors by Env, Vpu, and Nef proteins, which promote viral release while preventing reinfection by downregulating CD4 and co-receptors (3, 34, 35). Importantly, this exclusion is incomplete and strongly context-dependent: high MOI, cell-to-cell transmission, asynchronous entry, and ongoing viral replication within tissue reservoirs permit superinfection and same-cell coinfection *in vivo*, providing the substrate for recombination and the generation of diverse recombinant HIV-1 genomes.

Among pestiviruses, a primary infection with bovine viral diarrhea virus can prevent homologous superinfection within 30–60 minutes by blocking entry via E2 glycoprotein; even if a second genome enters, its replication is suppressed (36). For vaccinia virus (VV), a poxvirus, superinfecting virions can attach to the cell surface but are blocked at the membrane fusion stage by the viral proteins A56 and K2 (25, 27), thereby maintaining replication exclusivity (25). This is consistent with earlier cell culture studies showing resistance to superinfection in poxvirus-infected cells (28, 30). Alphaviruses can reduce susceptibility to secondary infection by receptor depletion and cytoskeletal remodeling, thereby limiting entry (29). In contrast, atypical porcine pestivirus (APPV) strikingly enhances susceptibility to classical pestiviruses through an entry pathway that appears independent of ADAM17, a host metalloprotease implicated in pestivirus entry, indicating divergent attachment or entry and explaining the absence of SIE in APPV-infected cells (37).

#### Replication and translation complex exclusion

At later stages of the viral life cycle, SIE can be enforced through exclusion from replication or translation complexes. HCV-infected cells resist superinfection by another HCV strain through a post-entry block at the RNA replication step; however, adaptive mutations in E1, p7, NS5A, and the 3′ UTR poly(U/UC) can circumvent this barrier, yielding chimeric populations that exemplify rapid within-host evolution (38).

#### IFN-mediated interference and partial failure of exclusion

SIE can be mediated either by intrinsic viral mechanisms or indirectly through host innate immune responses. In many systems, infection-induced interferon signaling establishes a refractory state that limits secondary infection. For severe acute respiratory syndrome coronavirus 2 (SARS-CoV-2), infection with one variant can restrict entry of another through a combination of spike occupancy, membrane remodeling, and interferon signaling (31). As in HIV-1, partial failure of SIE during periods of high viral circulation provides a powerful mechanism for rapid genetic innovation, immune escape, and the combination of advantageous traits.

Overall, SIE is best viewed as a virus-directed interference mechanism that stabilizes replication by limiting secondary infection of the same cell. In most settings, this reduces same-cell coexistence and restricts direct competition between viral genomes. However, exclusion is rarely absolute and can be incomplete or bypassed.

### Facilitative interactions: synergy, dependence, and complementation

Not all within-host interactions are antagonistic. Synergy refers to reciprocal enhancement between coinfecting viruses. However, many reported positive virus-virus interactions are better described as facilitation, in which one virus enhances the replication, spread, or pathogenic potential of another without clear evidence of reciprocal benefit. These effects can occur directly through viral gene products or indirectly through immune suppression, tissue damage, altered receptor expression, or remodeling of host pathways. For example, Epstein-Barr virus (EBV)-encoded nuclear antigen 1 (EBNA1) can enhance HCV replication (32, 39), while West Nile virus (WNV) or IAV infection has been reported to enhance replication of an insect-specific flavivirus or human parainfluenza virus type 2, respectively (40–42).

Dependence/assistance describes interactions in which a fully competent virus supports the replication of a defective virus, a so-called dependovirus. The canonical example is the HBV and hepatitis D virus (HDV) pair, which exemplifies parasitic dependence with antagonistic outcomes. HDV requires the HBV surface antigen (HBsAg) for virion assembly but suppresses HBV replication through transcriptional interference and strong interferon-stimulated gene (ISG) responses, thereby intensifying hepatic inflammation (43–45). Clinically, simultaneous HBV/HDV coinfection tends to cause acute, often self-limited hepatitis, whereas HDV superinfection in chronic HBV carriers accelerates fibrosis, cirrhosis, and hepatocellular carcinoma (46–48). The immune contribution is decisive: robust cytotoxic T-cell and type I IFN programs curb HBV but damage hepatocytes. Because both viruses are hepatotropic, the sequence of infection (HDV superinfection of chronic HBV) and the liver-specific tissue context together amplify immunopathology (45, 49–51). Recent work expands the virus families with which deltaviruses can interact to produce infectious particles to rhabdo-, herpes-, and arenaviruses (52). A comparable helper-dependent relationship is observed for adeno-associated virus, which requires coinfection with a helper virus such as adenovirus or herpesvirus to support productive replication. These systems illustrate how defective or satellite viruses exploit the structural or replicative machinery of a helper virus to complete their life cycles beyond experimental settings.

A related but distinct consequence of transient intracellular coexistence is complementation by pseudotyping, wherein virions incorporate heterologous envelope proteins and thereby broaden their host range. During coinfection, HIV-1 can be pseudotyped with envelope proteins derived from HIV-2, human T-cell lymphotropic virus 1 (HTLV-1), or even herpes simplex virus (HSV), generating chimeric virions with expanded tropism (53–55). Such pseudotypes can infect CD4-negative targets, including epithelial or keratinocyte populations, and may facilitate spread within anatomical compartments typically restrictive to HIV-1. This phenomenon is not restricted to retroviruses; respiratory viruses can also generate hybrid virus particles. Coinfection with IAV and RSV can result in viral particles containing ribonucleoproteins and glycoproteins from IAV and RSV (56). Although these events are rare *in vivo* for HIV and have not yet been documented for IAV/RSV hybrids, they illustrate how transient coinfection can yield novel viral particles with altered tissue tropism and potentially modified pathogenesis.

Together, antagonistic and facilitative interactions define whether viruses remain mutually exclusive, coexist transiently, or exchange functional components within the same cellular or tissue environment. While interference and SIE restrict opportunities for coexistence, dependence, complementation, and pseudotyping illustrate that coinfecting viruses can also exploit one another’s gene products or structural components. These transient states of coexistence provide the mechanistic basis for the evolutionary consequences of coinfection and superinfection.

### Evolutionary consequences of incomplete exclusion and transient coexistence

Coinfection and superinfection can have important evolutionary consequences when barriers to viral coexistence are incomplete, delayed, or bypassed. Although SIE limits secondary infection and thereby reduces opportunities for genetic exchange, transient coexistence of distinct viral genomes within the same cell or tissue can enable recombination, reassortment, complementation, or phenotypic diversification. Thus, SIE represents a balance between maintaining replication stability and allowing limited opportunities for diversification under specific ecological or selective conditions (57). This trade-off is also supported by computational modeling, which suggests that SIE can provide a strong short-term competitive advantage by preventing superinfection, while potentially limiting long-term adaptive potential by reducing the genetic diversity generated through multiple infection events (57). Moreover, this trade-off between restricting coinfection and permitting limited diversification is illustrated by alphavirus studies in which experimental disruption of SIE enhances superinfection and genetic recombination but compromises transmission stability, underscoring that natural SIE represents a balance between diversification and fitness (58–60). Plant and insect viruses further illustrate how transient coinfections can promote RNA recombination and segment reassortment, accelerating viral diversification but often at the cost of genome stability. IAV provides one of the clearest examples of evolutionary innovation through coinfection. Because IAV has a segmented genome, coinfection of the same cell by genetically distinct strains can enable reassortment, generating progeny with new combinations of genome segments. Coinfection of intermediate hosts, such as swine, can create opportunities for reassortment between genetically distinct influenza viruses, raising concerns that viruses adapted to non-human hosts may acquire properties that facilitate transmission in humans (16, 61).

For non-segmented RNA viruses, recombination provides a parallel route to diversification. Coronaviruses are particularly relevant because their replication strategy permits template switching during RNA synthesis. Coinfection and recombination within the same genus of porcine CoVs drive virus evolution (62). Recombination of different CoV strains from distinct lineages could expand the host range. A recent detection of recombinant porcine deltacoronavirus in patient plasma samples confirmed this notion (63). The detected porcine coronavirus arose from coinfection and recombination of China and SEA lineage viruses (62).

However, the emergence of successful recombinant lineages does not imply that productive genetic exchange is frequent at that level of individual infections. For CoVs, recombination requires that two viruses infect the same host, share sufficient tissue and cell tropism, and co-locate within compatible replication organelles (64). Accumulating genomic evidence demonstrates that same-cell coinfection with distinct SARS-CoV-2 variants occurs *in vivo* and enables frequent recombination, giving rise to recombinant lineages such as XBB and XFG. The true frequency of these events is likely underestimated, as only recombinants with sufficient sequence divergence and a selective advantage are reliably detected and persist at the population level. The SARS-CoV-2 pandemic, with its unprecedented sequencing effort, provides an opportunity to estimate the frequency of coinfections. Analysis of more than 2 million SARS-CoV-2 sequencing samples showed that coinfections between SARS-CoV-2 lineages accounted for 0.35% of the samples (65). This value likely underestimates the true frequency of coinfection, because transient, low-frequency, or non-productive coinfections may be missed by sequencing-based detection. Many coinfections may be transient, occur below the detection threshold, or fail to generate viable recombinant progeny. Nevertheless, during periods of intense co-circulation, even rare coinfection and recombination events can produce recombinant viruses that persist if they acquire a selective advantage. Thus, SARS-CoV-2 recombinants demonstrate how rare genetic exchange events can still have significant evolutionary and epidemiological consequences.

Experimental studies further support the idea that tissue structure and SIE can limit opportunities for genetic exchange. At the cellular and tissue level, SARS-CoV-2 can suppress secondary infection in a time-dependent manner and establish spatially segregated infection foci with minimal overlap *in vitro* (31). Such spatial structuring may restrict opportunities for recombination, helping to maintain distinct viral populations within infected tissues (31, 66). While such spatial restriction mechanisms cannot be directly inferred from sequencing data alone, they provide a plausible biological framework for interpreting patterns of limited detectable coinfection observed at the population level.

Like RNA viruses, large DNA viruses can also undergo substantial genome remodeling during evolution. Poxviruses provide an additional DNA virus example in which recombination and genome architecture can shape viral evolution. Although poxviruses have large dsDNA genomes and generally evolved differently from RNA viruses, their diversification is strongly influenced by homologous and non-homologous recombination, gene duplication, gene loss, horizontal gene transfer, and large-scale genome rearrangements. These processes can alter host range, immune evasion, virulence, and adaptation to new hosts. Thus, poxviruses illustrate that the evolutionary consequences of coinfection are not limited to RNA-virus recombination or influenza reassortment, but also include structural genome remodeling in large DNA viruses (67).

Together, these examples demonstrate that coinfection does not automatically lead to recombination or reassortment. Evolutionary outcomes depend on the same determinants that shape acute virus-virus interactions: timing, local viral burden, tissue and cell tropism, replication-site compatibility, innate immune activation, and the efficiency of exclusion mechanisms. Coinfection, therefore, creates an evolutionary trade-off; it can promote evolution and phenotypic diversity, but it can also compromise genome stability, replication efficiency, and transmission fitness.

## HOST- AND POPULATION-LEVEL OUTCOMES

At the host level, the cumulative effects of viral coinfection and superinfection extend beyond individual cells. Beyond cell-intrinsic interactions, viral coinfections reshape local and systemic immunity by modifying host susceptibility and response. Innate immune responses, such as IFNs, not only act within the infected cell to limit viral replication but can also influence neighboring cells and tissues, creating an antiviral state that shapes subsequent infections (68). Each viral encounter leaves an immunological imprint through cytokine profiles, trained innate responses, and memory T and B cells that influence responses to subsequent challenges (69–71). When a virus invades, this pre-existing immune landscape dictates whether the outcome will be protective, neutral, or exacerbated.

### Epidemiological evidence for virus-virus interaction

The co-circulation of viruses, especially respiratory viruses, leads to distinct global infection patterns. During the COVID-19 pandemic, circulation of RSV was markedly reduced or delayed in many regions (72, 73). A similar effect was previously observed only during the 2009 IAV pandemic. In the Northern Stockholm region, RSV historically exhibited a biennial outbreak pattern; however, this pattern was disrupted during 2009 (74). RSV infection was delayed and reduced, resulting in an altered outbreak cycle in subsequent seasons. Changes were attributed to viral interference by pandemic IAV p2009H1N1 and to concurrent shifts in population immunity and contact patterns. Furthermore, mathematical modeling of epidemiological data from Hong Kong and Canada suggests that both RSV and IAV can confer protection against infection with other viruses for up to five months after recovery (75) (Table 1).

**TABLE 1:** Summary of virus coinfection effects observed from epidemiological surveys^*a*^

Primary infection	Secondary infection	Outcome	Model system	Cells/host factors mediating interference	Reference
*Hepaciviridae*					
GBVC	HIV-1	Reduced the level of HIV-1 RNA	Epidemiological studies		(76)
HBV	HDV	HDV replication depends on HBV	Epidemiological studies	Viral protein (HBV HBsAg)	(43)
*Orthomyxoviridae*					
IAV (pmd09 H1N1)	RV(A16)	Suppression of RV circulation	Epidemiological survey	Host innate immunity	(77, 78)
*Picornaviridae*					
RV	IAV pmd09 H1N1	Suppression of IAV circulation	Epidemiological survey	Host innate immunity	(79–82)
RV	SARS-CoV-2	Reduction of risk for SARS-CoV-2 infection	Epidemiological survey	Host innate immunity	(78)
EVs	Oral polio (OPV) and rotavirus vaccine (RTV) (GlaxoSmithKline)	Reduced OPV and RTV immunogenicity	Epidemiological survey	–	(83, 84)
*Pneumoviridae*					
RSV	IAV (pmd09 H1N1)	Suppression of RSV circulation	Epidemiological surveys and modeling	Most likely host innate immunity and IFN response	(74, 75)
*Retroviridae*					
HIV-1	HPV	Promotion of HPV persistence	Epidemiological study		(85)

^*a*^ EVs, enteroviruses; GBVC, GB virus C; HBV, hepatitis B virus; HDV, hepatitis D virus; HIV-1, human immunodeficiency virus 1; HPV, human papillomavirus; IAV, influenza A virus; OPV, oral polio; RSV, respiratory syncytial virus; RV, rhinovirus; RTV, rotavirus vaccine; –, no data.

The spread of the IAV population can be interrupted by another virus, RV. Several studies in Australia, France, and the USA have examined the effect of RV circulation on the spread of IAV p2009H1N1 (79–81). These studies showed that RV infection reduces the likelihood of IAV infection, primarily through a strong induction of ISGs. The timing of RV infection relative to IAV may also play a significant role. Notably, RV was circulating widely in early autumn before p2009H1N1 spread in France, and it was suggested that it delayed the onset of the IAV epidemic (80). Another study, which used PCR-based detection of respiratory viruses in clinical samples, showed that RV detection was associated with a reduced likelihood of co-detection with other respiratory viruses, including IAV and human coronaviruses (79, 86). Conversely, IAV can also reduce RV infection. Analysis and modeling of epidemiological data collected over 9 years and approximately 36,000 individual samples indicated that IAV and IBV can negatively impact RV susceptibility and lead to asynchronous viral circulation (82). Mechanistic modeling consistent with these observations further indicated that longer-lasting antiviral refractory periods following primary infection substantially reduce superinfection risk, with estimated reductions of ~23% after 2 days and ~61% after 7 days (82). Consistent with this, IAV infection, particularly IAV p2009H1N1, was reported to reduce RV spread and the number of RV infections in a smaller study that conducted sequential testing of a cohort of schoolchildren (77). Beyond the interplay with IAV, RV infection was suggested to reduce the risk of SARS-CoV-2 infection (78). Epidemiological data suggest that RV infection within 30 days prior to SARS-CoV-2 infection may reduce SARS-CoV-2 viral load by up to 50% (78). However, the RV protective effect may be age-related, as children (<12 years of age) were more susceptible to RV and showed a stronger innate immune response following RV infection (78, 87) (Table 1). RV circulation was mostly unaffected by non-pharmaceutical measures used to control SARS-CoV-2 spread, which set RV apart from other respiratory viruses such as RSV, IAV, and IBV (88, 89). These observations illustrate how respiratory virus interactions can shape both individual susceptibility and population-level circulation patterns. However, such patterns must be interpreted cautiously because changes in viral circulation can also arise from broad perturbations of transmission dynamics.

At the population level, changes in viral circulation patterns can arise not only from intrinsic virus-virus interactions but also from broad perturbations of transmission dynamics. This was illustrated during the SARS-CoV-2 pandemic, when widespread non-pharmaceutical interventions were associated with dramatic reductions in influenza and other respiratory viruses, substantial disruptions to typical seasonal patterns, and the apparent disappearance of the IBV Yamagata lineage (88, 89). The IBV Yamagata lineage has been circulating globally for several decades since IBVs split into the Victoria and Yamagata lineages in the 1970s (90). Usually, the dominance of the Victoria and Yamagata lineages alternated from year to year. However, since the beginning of the SARS-CoV-2 pandemic in 2020, no IBV Yamagata isolates have been detected globally (91). It is not clear what led to the extinction of the Yamagata lineage. While direct viral interference cannot be excluded, the most likely scenario is that global non-pharmaceutical interventions implemented to limit SARS-CoV-2 transmission abruptly disrupted IBV Yamagata spread and reservoir maintenance, as there is no IBV reservoir in the animal population (92). Together, these observations illustrate how virus-virus interference and public health interventions can reshape viral population structure.

### Systemic immune imprinting and heterologous immunity

Systemic immune imprinting refers to the long-lasting shaping of the host immune landscape by an initial infection, which influences the magnitude, specificity, and quality of responses to subsequent infection. When a primary viral infection occurs, naïve T cells become activated, differentiate into effector T cells, and ultimately into memory T cells. Upon subsequent infection with a related virus sharing partially conserved epitopes, pre-existing immunity strongly shapes the outcome through heterologous immunity (3, 93–95). This phenomenon, well described in experimental and clinical studies, is primarily driven by cross-reactive T cells recognizing conserved epitopes between related viruses, as well as by bystander activation of memory T cells, and particularly during simultaneously or closely spaced infections, by cytokine-mediated remodeling of the local immune landscape. Heterologous immunity has been observed mainly between related viruses (e.g., influenza strains) but can also occur between more distantly related and unrelated viruses (e.g., prior lymphocytic choriomeningitis virus [LCMV] immunity enhances or alters the response to vaccinia or Pichinde virus), and even across viruses and non-viral pathogens (94, 95). The outcomes can be beneficial (cross-protection) or detrimental (immunopathology) (96–98), depending on factors such as viral type (98), infection dose (97), stage of infection or timing (96), and host age (99). Together, these observations highlight the central role of T cell-mediated mechanisms in virus-virus interactions, while also implicating additional immune cell populations, including innate lymphoid cells, NK cells, dendritic cells, and macrophages, which modulate cytokine production, antigen presentation, and tissue inflammation. For a more detailed discussion of T cell-driven heterologous immunity and immune imprinting, we refer the reader to recent comprehensive reviews (95, 100–102).

### Order and timing of infection as a determinant of outcome

Timing and sequence are among the strongest determinants of the outcome of viral coinfection and superinfection. Sequential exposure to distinct viruses can reprogram responses through innate immune priming, IFN-mediated antiviral states, altered antigen presentation, and tissue-specific trafficking of effector T cells, thereby shaping susceptibility, protection, or immunopathology during subsequent infections (103). In some settings, a first infection induces a transient antiviral state that protects against a second pathogen, whereas in others, prior infection redirects immune cells to inappropriate anatomical compartments, impairing local immune control and exacerbating disease. Mechanistically, these outcomes can involve shifts in immunodominance hierarchies, remodeling of the TCR repertoire, cross-reactive T-cell responses, and cytokine-mediated changes locally (95, 104).

The timing of secondary infection relative to innate activation is equally critical. A second virus entering before ISGs are fully expressed may establish replication, whereas infection after ISG induction typically fails (14). This temporal gating explains why altering the interval between infections can dramatically shift disease outcomes. Differences in pattern recognition receptor engagement (e.g., RIG-I, MDA5, TLRs, CLRs, or cGAS-STING pathway for DNA viruses) further refine susceptibility windows and influence downstream inflammatory and pathological outcomes (14, 105, 106). A prominent example is IAV-RSV superinfection. Prior IAV infection can attenuate subsequent RSV disease (11). In mice, RSV infection followed by IAV infection 2 days later led to delayed and milder disease compared to simultaneous RSV/IAV infection. In contrast, RSV administration 2 days after IAV exacerbates IAV disease (11, 107–109), consistent with a protective role of early innate immune activation. Sequential infections targeting different organs can also promote pathological immune misdirection. Infection with SFV followed by IAV resulted in aberrant recruitment of IAV-specific CD8^+^ T cells to the brain rather than the lung, impairing pulmonary responses, elevating lung inflammation, and aggravating pathology. This pathology was associated with suboptimal CD8^+^ T-cell priming and antigen-presenting cell paralysis (110). Conversely, when IAV infection preceded SFV, systemic induction of type I IFN attenuated SFV replication, reducing infectious SFV titers and inflammatory cytokine and chemokine production in the central nervous system (CNS) (111). Similarly, IAV infection followed by DENV-2 impaired host immune responses, driving uncontrolled DENV replication and severe lung damage due to blunted lung transcriptional responses and reduced monocyte recruitment (112). Reversing the order, with DENV infection preceding IAV, resulted in milder disease. Together, these examples highlight that infection sequence critically shapes immune outcomes and disease severity.

Simultaneous coinfections can also alter immunity, although outcomes are often less predictable. In a mouse model, coinfection with ectromelia virus (ECTV, mousepox) and LCMV reduced ECTV viral load and ameliorated ECTV-induced disease compared to the single infections (Table 2) (113). This protective effect was mediated by LCMV-induced type I IFN that overcomes ECTV-mediated type I IFN suppression (11). Protection may arise from innate immune activation (114), bystander CD4^+^/CD8^+^ T function (99), and, in some settings, cross-reactive CD8^+^ T cells (115). However, other viral combinations can worsen immunity: IAV-immune mice are protected against VV but not against murine cytomegalovirus (MCMV) or LCMV. Such effects are often non-reciprocal, with one virus dominating the interaction, often through IFN signaling, altered immune priming, or competition for limiting cellular resources (104).

The local MOI and innate activation threshold further modulate these timing-dependent effects. Although uniformly high MOI across tissues is unlikely *in vivo*, locally elevated viral burden can occur at sites of intense replication, such as infection foci, lymphoid tissues, or during cell-to-cell transmission. Under these conditions, multiple viral genomes may enter the same cell or neighboring cells within a short time window, accelerating replication kinetics, increasing the likelihood of defective particle formation, and amplifying IFN responses. For IAV, increasing MOI enhances replication and elevates type III IFN (IFN-λ) without necessarily increasing yield in human airway cells, demonstrating cell type-specific coupling between coinfection and innate activation (116).

Thus, infection order, interval, local viral dose, and innate immune thresholds should be viewed as interconnected determinants rather than separate variables. Together with tissue tropism and baseline immune state, they define whether viral encounters lead to interference, facilitation, immune protection, or immunopathology.

## VIRAL COINFECTIONS AT TISSUE LEVEL: CELLULAR CONTEXT SHAPES VIRUS-VIRUS INTERACTIONS AND OUTCOMES

Viral coinfections at the tissue level are strongly constrained by viral tropism because direct interactions require viruses to reach the same anatomical site and, in some cases, the same cell type.

Tissue and cell-type specificity therefore limit potential coinfection partners and determine whether interactions occur at the intracellular, through paracrine signaling, or in spatially segregated levels. Even within permissive tissues, heterogeneous receptor expression (117, 118) and local innate immune signaling can restrict viral spread, leading to the formation of infection foci (119).

Advances in single-cell transcriptomics, spatial profiling, and primary tissue models such as organoids and air-liquid interface cultures have substantially improved our understanding of virus tropism and cell-type-specific susceptibility. In the respiratory tract, for example, SARS-CoV-2 and influenza viruses depend on distinct entry factors that are unevenly distributed across epithelial cells. Viral entry factors such as ACE2 and sialic acid follow certain expression profiles. ACE2 expression is enriched in the upper respiratory tract and is highest in ciliated cells, whereas basal cells express comparatively low levels (120), while the opposite is true for α2-3 sialic acid expression (121, 122). Detailed discussions of tissue tropism across the gastrointestinal, respiratory, and nervous systems are available (123–125). Below, we focus on how tissue context shapes virus-virus interactions in several anatomical sites.

### Respiratory virus coinfections in the airway epithelium—tropism and innate immunity

During the SARS-CoV-2 pandemic, concern arose that coinfections with common respiratory viruses, such as IAV, IBV, RV, and RSV, could lead to more severe pathology and higher mortality (Table 2). This concern was consistent with earlier within-host modeling studies showing that infection timing, inoculum size, target cell availability, and cellular coinfection can shift the outcome of respiratory virus interactions from coexistence to suppression (126–129). More recent experimental work using airway epithelial cultures and organoids has begun to define how SARS-CoV-2 homo- and hetero-coinfections are shaped by tissue context, cell-type susceptibility, local MOI, and innate immune responses (129).

IAV and SARS-CoV-2 were shown to have a synergistic effect on each other’s tropism. IAV infection has been reported to increase ACE2 transcriptional levels, whereas the SARS-CoV-2 Delta variant increased the levels of α2-3 sialic acid on the surface of induced pluripotent stem cell hiAT2 alveolar organoids and bronchial epithelium (130, 131). However, this apparent reciprocal effect on entry-factor expression does not necessarily translate into altered viral replication in all models. For example, in lung tissue explants, coinfection did not reduce infection by SARS-CoV-2 or IAV. In contrast, some IAV strains, such as H5N1, can suppress SARS-CoV-2 infection, particularly in the superinfection setting. The limiting effect of IAVs on SARS-CoV-2 can be strain-specific and depends on the intrinsic features of the IAV strain, as less pathogenic H3N2 strains do not suppress SARS-CoV-2 infection to the same extent as H1N1 (132). Different impacts of virus coinfections were observed across different tissue model systems in cell type prevalence and cell susceptibility to viruses. Several IAV strains (H3N2 and p2009 H1N1) were shown to suppress SARS-CoV-2 variant superinfection in nasal and bronchial epithelium models due to induction of a strong type I IFN response (133–135). SARS-CoV-2 can also exhibit homologous or variant-level SIE in cultured cells: once primary infection is established, secondary infection by a different variant is strongly reduced (26, 31). Similarly, reports indicate that prior SARS-CoV-2 infection can reduce the replication efficiency of IAVs during superinfection (133).

Similarly to IAVs, IBV infections have been reported to reduce superinfection by SARS-CoV-2 variants *in vitro* and in *ex vivo* airway models (23, 136). However, there are SARS-CoV-2 variant-dependent factors as in the case of IAV. While Omicron EG.5.1 appears to be less affected by prior IBV infection than earlier variants, the available data are limited and do not allow definitive conclusions regarding enhanced competitive fitness (136).

At the tissue level, IBV lineages replicate efficiently in human nasal epithelial cells and share tropism for ciliated epithelial cells, yet they display distinct preferences for specific epithelial subpopulations (137). IBV Victoria shows a modest enrichment in MUC5AC^+^ goblet cells, whereas IBV Yamagata preferentially infects progenitor basal cells (138). This fine-scale partitioning within the nasal epithelium illustrates how closely related viruses can occupy overlapping but non-identical cellular niches within the same tissue.

Influenza viruses can also suppress superinfection by another coronavirus. For example, human coronavirus OC43 (CoV-OC43) did not significantly affect IAV or IBV replication, whereas CoV-OC43 titers were reduced in the presence of influenza viruses in bronchial epithelial cells (139). However, a synergistic effect was observed in human primary small airway epithelial cells, where CoV-OC43 infection increased IAV replication and vice versa (140). Importantly, these observations reflect cell- and tissue-level interactions.

As discussed before, numerous reports show that influenza viruses can suppress coronaviruses. This is not the case for all respiratory viruses. For RSV, SARS-CoV-2 suppresses RSV release of infectious virus in coinfection and superinfection settings, whereas RSV infection does not negatively impact SARS-CoV-2 replication in nasal epithelial cells (133). In bronchial epithelial models, however, RSV production of infectious particles was suppressed by SARS-CoV-2 infection in a coinfection setting (134). Overall, the relationship between SARS-CoV-2 and RSV is more dependent on the sequence of infection than on virus type-specific features. It was observed in the mouse model setting that SARS-CoV-2 suppressed RSV RNA replication, and vice versa, in BALB/c mouse lung tissue in a sequential infection setup (141). The overall effect of SARS-CoV-2 and RSV interaction can depend on timing, and viruses can affect different stages of their life cycle. RSV was also shown to suppress another human respiratory virus, human metapneumovirus (HMPV), which frequently infects the lower respiratory tract. RSV suppressed HMPV infection in air-liquid interface airway models at a superinfection setting. This phenotype was mediated by high levels of type III IFN induced by RSV infection and by IFN-mediated suppression of HMPV replication (142). RSV interactions with other viruses are dependent on the infection sequence and the type of virus coinfecting a cell.

Mechanistic studies using human bronchial epithelial cells showed that RV and SARS-CoV-2 coinfections severely disrupted SARS-CoV-2 infection. In addition, an adverse RV effect was observed in the superinfection setup, too. RV infections were shown to diminish SARS-CoV-2 independent of infection sequence in relation to SARS-CoV-2 (127, 143, 144). However, different observations were obtained in murine lung epithelial cells using the model coronavirus murine hepatitis virus strain 1 (MHV-1). RV replication did not disrupt MHV-1 replication in lung epithelial cells, and a fraction of cells expressed proteins from both viruses in a superinfection setting (145). Furthermore, RV is very efficient at inducing an IFN response, which can also limit the replication of other enveloped viruses, such as IAV and IBV, in human airway epithelium models (81, 146). Increased levels of type I IFN induced by primary RV infection were also shown to limit subsequent RV infections 5 days after the initial infection in lung endothelial cells (147). In summary, RV can suppress several types of viruses during coinfection.

Beyond classical respiratory viruses, enteroviruses such as coxsackievirus A7 and enterovirus A71 also negatively affected SARS-CoV-2 replication in the respiratory tract. Prior infection with both enteroviruses led to severe suppression of SARS-CoV-2 superinfection. The adverse effect of enteroviruses on SARS-CoV-2 replication was mediated by inducing high levels of type I and II IFN and proinflammatory cytokines (148). Overall, these findings indicate that diverse viral infections capable of inducing a strong antiviral state within the respiratory epithelium can suppress SARS-CoV-2 replication, highlighting the dominant role of tissue context and innate immune signaling in shaping virus-virus interactions.

### Vector-borne viruses: skin, systemic spread, and CNS involvement

Beyond the respiratory tract, similar principles of interference and facilitation apply to vector-borne viruses. However, the dominant mode of interaction *in vivo* is likely sequential exposure rather than true simultaneous coinfection. Although mosquito-transmitted arboviruses such as DENV, chikungunya virus (CHIKV), and Zika virus (ZIKV) co-circulate in many endemic regions, simultaneous infection of the same host with multiple arboviruses is less common than repeated exposure over time (149, 150). This creates a pre-existing immune and antibody landscape that can shape subsequent infections. As a result, prior exposure to arboviruses or serotypes over time can generate a pre-existing immune and antibody landscape that shapes subsequent infection outcomes.

Experimental coinfection and superinfection studies provide important mechanistic insights (Table 2). DENV suppresses ZIKV infection in human dermal fibroblast models (151). This effect is associated with induction of type I and II IFNs, upregulation of several ISGs and chemokines, and downregulation of the transcriptional levels of the ZIKV receptor tyrosine-protein kinase receptor 3 and additional genes associated with clathrin-mediated endocytosis (151). In contrast, ZIKV infections did not affect DENV viral RNA levels when both viruses were coinfected (151). ZIKV has also been shown to activate autophagy pathways to limit superinfection by downregulating the phosphatidylserine receptors AXL receptor tyrosine kinase (AXL) and T-cell immunoglobulin and mucin domain (TIM-1) (152).

**TABLE 2:** Summary of virus coinfection data from experimental studies^*a*^

Primary infection	Secondary infection	Outcome	Model system	Cells/host factors mediating interference	Reference
*Arenaviridae*					
LCMV	PV, VV	Reduced replication of PV and VV	Murine models	CD4- and CD8-positive cells	(94)
LCMV (Armstrong and clone-13 strains)	ECTV	Ameliorated ECTV disease	Murine models	Innate immunity, type I IFN	(113)
LCMV (Armstrong strain)	MCMV (Smith strain)	Exacerbated MCMV disease	Murine models	Adaptive immunity	(113)
*Coronaviridae*					
SARS-CoV-2 (BA.5)	LIAV (quadrivalent)	Reduced replication of LAIV	Human primary cells	Innate immunity	(136)
SARS-CoV-2 (D614G, Delta, Omicron)	IAV (pdm09 H1N1 and H3N2)	Enhanced IAV pdm09 H1N1 replication and IAV infection is suppressed	Human primary cells	Innate immunity induced at different levels by different SARS-CoV-2 variants	(23, 130, 132–135)
	IBV (Yamagata and Victoria) and vaccine strains	Effect on IBV depends on the SARS-CoV-2 variant infection	Human primary cells and cell lines	Innate immunity	(127, 136)
SARS-CoV-2	RSV (A2)	Suppressed RSV replication	Human primary cells	Probably innate immunity	(133)
SARS-CoV-2	RSV	Suppressed RSV replication	Murine models	Innate immunity	(141)
MHV1	IAV (PR8)	Suppressed IAV infection	Murine models	Type I IFN response	(24)
*Flaviviridae*					
HCV	HBV	HBV infection suppressed	Cell line	Viral protein (HCV core)	(17, 20, 46)
HCV	Homologous virus infection	Homologous virus superinfection prevented	Cell line	Viral protein (E1, p7, NS5A) and genome 3′ UTR, the poly(U/UC) tract	(38)
DENV	ZIKV	ZIKV infection was suppressed	Human primary cells	Innate immunity	(151)
DENV	CHIKV	CHIKV infection was suppressed	Human primary cells	Innate immunity	(153)
DENV (D220)	IAV (pmd09 H1N1)	IAV disease less severe	Murine models	–	(112)
WNV	CxV	WNV infection increased the likelihood of CxV infection	Mosquitoes	–	(40, 41)
Louping ill virus	YFV (French neurotropic vaccine strain)	No effect on YFV replication	Cell line	–	(28)
APPV	Pestiviruses	Increased susceptibility of cells to pestiviruses	Cell line	Primary infection might induce host cell membrane remodeling	(37)
BVDV	BVDV	Homologous virus superinfection prevented	Cell line	Viral protein (BVDV E2)	(36)
*Hepadnaviridae*					
HBV	HDV	HDV replication depends on HBV	Primary human cells, humanized murine model	Viral protein (HBV HBsAg)	(43, 49)
*Orthoherpesviridae*					
HSV-1	PRV	PRV inhibits entry or fusion, HSV-1 suppresses post-entry steps	Cell line	–	(29)
HSV-2	HIV-1	HSV promotes HIV-1 replication	Human primary cells	Proinflammatory response	(154)
EBV	HCV	HCV replication enhanced	Cell line	Viral protein (EBV EBNA1)	(32)
HCMV	HHV-6	Increased replication of HHV-6	Human primary cells	Proinflammatory response	(155)
VZV	HSV-1	Primary infection suppresses secondary infection, depending on infected cell type	Human primary cells	Cell type-specific expression of attachment factors	(156)
*Orthomyxoviridae*					
IAV (H1N1)	RV (A16)	RV infection suppressed, no effect on IAV	Human primary cells	Type I and III IFN	(23)
IAV (H1N1)	PIV (type 2)	Enhanced IAV infection	Cell lines	Increased cell fusion by PIV HN and F proteins	(42)
IAV (pmd09 H1N1)	DENV (D220)	DENV infection is exacerbated	Murine models	–	(112)
IAV (X31 H3N2)	SFV (A7 74)	SFV infection was attenuated	Murine models	Type I IFN levels	(111)
IAV/IBV	hCoV-OC43	IAV/IBV reduced	Human primary cells		(139)
*Picornaviridae*					
RV (1B)	IAV (PR8)	IAV infection suppressed	Murine models	Inflammatory response toward IAV	(24)
RV	SARS-CoV-2	Suppression of SARS-CoV-2 infection	Human primary cells	Innate immunity	(127, 143, 144)
RV	MHV-1	MHV-1 infection was not suppressed	Murine models		(145)
CVA7 (LEV-8 strain), EV A71	SARS-CoV-2	Decrease SARS-CoV-2 infection	Cell line and murine models	Innate immunity	(148)
*Pneumoviridae*					
RSV (A)	RV (A16)	RV infection suppressed, no effect on RSV	Human primary cells	Type I and III IFN	(23)
RSV (A2)	IAV (pdm09 H1N1)	IAV disease was aggravated	Murine models	Increase in severity of pulmonary symptoms	(107–109)
RSV	SARS-CoV-2	Suppression of SARS-CoV-2 replication	Human primary cells	Innate immunity	(134)
RSV	HMPV	Suppression of HMPV replication	Human primary cells	Innate immunity	(142)
*Poxviridae*					
VV	Homologous virus infection	Homologous virus superinfection prevented	Cell line	Viral protein (A56, K2)	(25, 27)
*Retroviridae*					
Retrovirus vectors	Homologous virus infection	Limited coinfection	Cell line	–	(30)
HIV-1	HIV-1	Primary infection suppresses secondary infection	Human primary cells	Viral NEF protein downregulates CD4	(34)
HIV-1	EBV	Reactivation of latent virus pools	Human primary cells		(157, 158)
HIV-1	KSHV	Reactivation of latent virus pools	Cell line		(159, 160)
HIV-1	HSV-2	HIV-1 promotes HSV reactivations	Cell line		(161, 162)
HIV-2	HIV-1	HIV-2 suppress HIV-1 infection	Human primary cells	HIV-2 protein Vpx	(163, 164)
HTLV-1/2	HIV-1	Pseudotyped particle formation	Human primary cells		(55, 165)
*Togaviridae*					
CHIKV	DENV	DENV infection was suppressed	Cell line	Innate immunity	(166)
SFV (A7 74)	IAV (X31 H3N2)	IAV infection was exacerbated	Murine models	CD8-positive cells	(110)

^*a*^ APPV, atypical porcine pestivirus; BVDV, bovine viral diarrhea virus; CHIKV, chikungunya virus; CVA7, coxsackievirus A7; CxV, Culex flavivirus; DENV, dengue virus; ECTV, ectromelia virus; EV A71, enterovirus A71; EBV, Epstein-Barr virus; HBV, hepatitis B virus; HCV, hepatitis C virus; HDV, hepatitis D virus; HSV-1, herpes simplex virus 1; HSV-2, herpes simplex virus 2; HCMV, human cytomegalovirus; HHV-6, human herpesvirus 6; HIV-1, human immunodeficiency virus 1; HMPV, human metapneumovirus; HTLV-1/2, human T-lymphotropic virus types 1 and 2; hCoV-OC43, human coronavirus OC43; IAV, influenza A virus; IBV, influenza B virus; KSHV, Kaposi's sarcoma-associated herpesvirus; LAIV, live attenuated influenza vaccine; LCMV, lymphocytic choriomeningitis virus; MHV1, mouse hepatitis virus 1; MCMV, murine cytomegalovirus; PIV, parainfluenza virus; PV, Pichinde virus; PRV, pseudorabies virus; RSV, respiratory syncytial virus; RV, rhinovirus; SFV, Semliki Forest virus; SARS-CoV-2, severe acute respiratory syndrome coronavirus 2; VV, vaccinia virus; VZV, varicella-zoster virus; WNV, West Nile virus; YFV, yellow fever virus; ZIKV, Zika virus; –, no data.

Context-dependent outcomes have also been observed for CHIKV-DENV interactions. In Huh7.5 liver cells, CHIKV suppresses DENV replication in superinfection and coinfection settings, possibly because CHIKV induces higher type I IFN activity than DENV (153, 166). However, the direction of the interaction can differ by cell type: CHIKV reduces DENV replication in Huh7.5 cells, but has the opposite effect in human peripheral blood mononuclear cells (PBMCs). DENV was shown to suppress the replication of infectious CHIKV, but coinfection with replication-capable CHIKV increased DENV viral titers (153). It was proposed again that the adverse effect of DENV infection on CHIKV replication was mediated by increased CHIKV sensitivity to type I IFNs (153). These observations indicate that arbovirus interactions depend not only on infection order and innate immune activation but also on the infected cell type.

Arbovirus interactions can also be shaped by systematic innate immunity and organ-specific susceptibility. CHIKV, DENV, and ZIKV can cause systemic disease, and some related viruses such as SFV are known to cause neurological complications such as encephalitis (Table 2). Previous work showed that the antiviral state caused by viral infection in different tissues can lead to a protective effect in the CNS. The infection with respiratory viruses, such as IAV, attenuated the severity of SFV infection in the CNS. Primary IAV infection induced robust type I IFN responses that establish a systemic antiviral state, likely restricting SFV replication and dissemination and thereby limiting viral spread to the CNS and subsequent neuropathology (111). In addition to IFN-mediated effects, intrinsic viral mechanisms can constrain superinfection. During WNV infection, non-structural viral proteins compete for limiting host factors, reducing their availability to incoming viral genomes and restricting secondary infection (167).

Together, these studies highlight that arbovirus coinfection outcomes emerge from the combined influence of infection order, innate immune activation, antibody landscapes, tissue-specific susceptibility, and cell-type-specific permissiveness. While *in vitro* and *ex vivo* systems are valuable for dissecting intrinsic viral interference, *in vivo* outcomes are likely dominated by sequential exposure and immune imprinting rather than frequent simultaneous same-cell coinfection.

### Mucosal, skin, and neuronal contexts of DNA virus coinfection

Mucosal tissues provide an additional context in which viral coinfections and superinfections are shaped by tissue architecture, cell-type composition, and baseline innate immune activity. Chronic DNA viruses that establish latency in mucosal tissues frequently encounter one another, creating opportunities for interaction that are distinct from acute RNA virus infections (Table 2) (168).

Human cytomegalovirus has been shown to support replication of human herpesvirus 6 (HHV-6) in coinfected skin dermal fibroblasts, resulting in increased viral DNA abundance compared with single infections (155). Another type of virus shown to coinfect was alphaherpesviruses, such as varicella-zoster virus (VZV) and HSV-1. These viruses exhibit different degrees of superinfection suppression depending on host cell type. In human foreskin fibroblasts, primary infection with VZV and HSV-1 strongly inhibited secondary infection by the other virus in a superinfection setting (156). This is consistent with alphaherpesvirus SIE involving altered receptor availability and interference with virion trafficking, with both processes being modulated by MOI and timing (29, 169). However, this SIE was not observed in a simultaneous coinfection setting. Furthermore, the adverse effects of primary infection on the secondary follow-up infection were mitigated in neuronal-type cells. In contrast, VZV and HSV-1 were able to infect the same neuronal lineage cells (156). The differential effectiveness of SIE in fibroblast and neuronal lineage cells may be related to differences in the expression of host innate immune factors or factors involved in viral replication.

These observations illustrate that DNA virus coinfection outcomes are strongly cell-type dependent: exclusion can be robust in fibroblasts but reduced or absent in neuronal-lineage cells, allowing coexistence under specific tissue conditions. To illustrate principles of co- and superinfection involving a chronic viral infection, we next focus on HIV-1, which provides a paradigmatic model for studying coinfection and superinfection at both cellular and clinical levels.

### HIV-1 as a chronic model for virus-virus interactions

HIV-1 provides a useful chronic model for virus-virus interactions because it establishes persistent infection, long-lived cellular reservoirs, and overlaps biologically and clinically with other lymphotropic and mucosal viruses. Clinically, HIV-1 coinfection refers to acquisition of phylogenetically distinct HIV-1 strains before seroconversion, including infection by multiple transmitted/founder viruses (170), whereas superinfection denotes acquisition of a new strain after seroconversion (171). At the cellular level, superinfection refers to infection of a cell that already harbors integrated proviral DNA (either transcriptionally active or silent) from a prior viral infection (35, 172). These events generally involve either multiple HIV-1 strains and, less commonly, HIV-1 and HIV-2 within the same cell, rather than completely unrelated viruses (53, 171, 173). Because HIV-1 persists in CD4^+^ cells and continuously reshapes the host immune environment, it provides an informative example of how viral interference, facilitation, and occasional complementation can coexist within the same chronic infection.

In chronic infections such as HIV-1, persistent low-level replication within tissue reservoirs generates a mosaic of permissive and refractory cells. Local heterogeneity in receptor availability and basal innate state dictates which cells can support superinfection and which remain resistant, effectively structuring viral populations at the microscale. This microscale heterogeneity shapes viral population structure and helps explain why HIV-1 can illustrate several core outcomes of virus-virus interaction, including SIE, recombination, complementation, and immune-mediated facilitation.

#### Genetic diversity, recombination, and superinfection exclusion

A hallmark of HIV-1 is its extraordinary genetic variability (quasispecies). The pandemic HIV-1 group M comprises multiple subtypes, numerous circulating recombinant forms, and unique recombinant forms, reflecting repeated dual infection and recombination between divergent strains (174, 175). At the same time, HIV-1 also displays a form of superinfection exclusion, often referred to in the retrovirus literature as superinfection resistance. In productively infected cells, Env-, Vpu-, and Nef-dependent downregulation of CD4 and co-receptors reduces susceptibility to reinfection of the same cell while promoting viral egress (33, 34). Additional mechanisms, such as co-receptor modulation, non-cytotoxic CD8^+^ T cells, and the CD8^+^ T-cell antiviral factor, can suppress HIV transcription by inhibiting Tat-mediated transcription from the HIV long terminal repeat (LTR) promoter (176).

Importantly, under conditions of ongoing replication, high local multiplicity, or efficient cell-to-cell spread, the same-cell coinfection can still occur, allowing integration of more than one provirus and creating opportunities for recombination and diversification (33, 53, 171, 173). Recent work further supports this concept that intact HIV-1 can superinfect reservoir cells carrying defective proviruses in people with non-suppressible viraemia, thereby promoting replication and diversification of defective genomes (177). This provides an *in vivo* example of how superinfection can rescue or amplify otherwise defective viral genomes, with implications for reservoir complexity, persistent viraemia under antiretroviral therapy (ART), and therapeutic strategies aimed at eliminating or silencing HIV reservoirs (177).

#### Intersubtype and cross-species interactions

Interactions with related retroviruses further illustrate how HIV-1 can participate in both antagonistic and complementary relationships. *In vitro*, HIV-1 and HIV-2 can coinfect the same cell, with HIV-2 selectively inhibiting HIV-1 replication in a dose-dependent and nonreciprocal manner (163). HIV-2 is a related lentivirus to HIV-1 that causes a generally milder disease compared to HIV-1 (164, 178). Mechanistically, HIV-2 suppresses HIV-1 through transcriptional interference at the HIV-1 LTR promoter, where the HIV-2 TAR acts as a decoy for Tat proteins (179). Nonetheless, reciprocal superinfection is possible: HIV-1 can superinfect HIV-2–infected cells, generating pseudotyped virions in which the HIV-1 genome is packaged within an HIV-2 envelope, thereby altering envelope-mediated entry properties and potentially modifying co-receptor usage (e.g., between CCR5 and CXCR4-tropic variants) (53, 178).

Coinfection with human T-cell lymphotropic viruses (HTLV-1/2) illustrates similar bidirectional influences. Although HTLV infection is relatively rare globally, HIV/HTLV coinfections can occur at appreciable frequencies in certain regions and high-risk populations, due to shared transmission routes and CD4^+^ T-cell tropism. Clinically, HIV-1/HTLV-1 coinfection is associated with enhanced disease progression, whereas HIV-1/HTLV-2 coinfection correlates with slower HIV-1 progression (180). Experimental studies have demonstrated that HIV-1 and HTLV-1 can coinfect the same CD4^+^ T cell *in vitro,* resulting in pseudotyping HIV-1 virions with HTLV-1 Env (55). Similarly, reciprocal pseudotyping has been observed, in which HTLV-1 particles incorporate HIV-1 Env. These chimeric virions are replication-competent, can compete with WT HIV-1, and may contribute to superinfection exclusion (55). Incorporation of HTLV-1 Env can alter HIV-1 entry properties by allowing HIV-1 genomes to enter cells through HTLV-1 Env-mediated pathways rather than canonical HIV-1 Env/CD4/co-receptor-dependent entry, thereby expanding the range of experimentally permissive cells. *In vivo*, sexually transmitted pseudotyped HIV-1 and HTLV-1, both coming from dually infected patients, may increase the chances of infecting intraepithelial CD4^+^ T cells, macrophages, and DCs, thereby enhancing viral spread and transmission potential (165). However, most evidence for same-cell coinfection and formation of pseudotyped or hybrid virions comes from *in vitro* or *ex vivo* systems using high multiplicities of infection, which facilitate these events under experimental conditions. Although HIV-1 pseudotyped with HTLV-1 Env has been detected in blood from HIV-1/HTLV-1-coinfected individuals and can infect primary epithelial cells, the frequency and functional significance of same-cell coinfection *in vivo* remain poorly quantified.

#### HIV and other persistent viruses

Beyond related retroviruses, HIV-1 frequently facilitates other persistent viruses through CD4^+^ T-cell depletion, cytokine remodeling, and impaired mucosal or immune control. HSV-1 and HSV-2 share epithelial tropism with HIV-1, and, as it is common for sexually transmitted mucosal infections, infection with one virus can facilitate acquisition or replication of another. HSV infection promotes local inflammation, epithelial cell disruption, and mucosal lesions or microabrasions, thereby lowering barrier integrity and enhancing HIV-1 infection. In addition, the associated immune response leads to the accumulation of activated CD4^+^ T cells and other target cells at mucosal sites, which are preferentially infected by HIV-1 (154). Conversely, HIV-1 infection enhances HSV acquisition, reactivation, and disease severity (181–183), while HSV increases HIV-1 replication through inflammatory signaling pathways (54, 161, 162). Thus, facilitation in this setting reflects both barrier disruption and inflammatory target-cell recruitment.

Other herpesviruses illustrate related but non-identical mechanisms. β-Herpesviruses, like HHV-6 and HHV-7, infect CD4^+^ T cells, thereby enhancing HIV-1 pathogenicity in coinfected individuals (184) and act as opportunistic cofactors in immunocompromised individuals (184, 185). Both viruses are able to infect CD4^+^ T cells *in vitro* and *in vivo*, with HHV-6 showing a preference for activated T cells. HHV-6 uses CD46 as a cellular entry receptor (186) and can transactivate the HIV-1 LTR (synergizing with Tat) (187). In addition, HHV-6 induces aberrant CD4^+^ on CD8^+^/NK cells, thereby modifying HIV-1 entry properties and co-receptor usage rather than enabling productive infection of these cells, and modulates T-cell receptors (CD3, CD46), contributing to T-cell function dysregulation (186). Another important effect of HHV-6 infection is the induction of RANTES, which inhibits CCR5 HIV-1 replication, promoting the switch to CXCR4 HIV-1 viruses (188). HHV-7, on the contrary, downregulates CD4 without increasing RANTES and suppresses replication of CCR5 but not CXCR4-tropic viruses in coinfected lymphoid tissue, leading to a faster HIV-1 progression in the absence of therapy. Together, these examples indicate that facilitation in chronic infection often reflects a combination of immune dysregulation, altered receptor landscapes, and local tissue context rather than a single shared mechanism.

#### HIV and oncogenic DNA viruses

HIV infection also amplifies the clinical consequences of oncogenic and persistent DNA viruses. HIV-driven immunosuppression facilitates reactivation and expansion of latent EBV and Kaposi’s sarcoma-associated herpesvirus reservoirs, thereby predisposing to lymphoproliferative disease, Kaposi’s sarcoma, primary effusion lymphoma, and the plasmablastic variant of multicentric Castleman disease, which are significantly more common in HIV-infected individuals (157, 159, 189). Although reciprocal transcriptional activation between HIV and herpesvirus gene products has been demonstrated *in vitro*, the dominant *in vivo* driver appears to be HIV-associated immunodeficiency rather than direct same-cell transactivation (157, 159, 189).

Similarly, HIV infection is associated with enhanced human papillomavirus (HPV) persistence and progression to malignancy in mucosal tissues, largely because HIV-induced immunodeficiency impairs HPV-specific T-cell responses, even in individuals with relatively preserved CD4^+^ T-cell counts (85). Additional factors, including increased exposure to multiple HPV types and altered sexual behavior, may contribute to higher HPV prevalence and persistence in HIV-positive populations. In hepatitis C (HCV) coinfection, HIV-associated immune dysregulation accelerates fibrosis and worsens treatment outcomes, while HCV-driven interferon and inflammatory responses can restrict or modulate HIV replication (38, 190, 191). The order and timing of infection again prove critical: immune reconstitution following ART may unmask hepatitis through immune reconstitution inflammatory syndrome-like flares, whereas HCV superinfection in HIV-positive individuals occurs in an immunologically exhausted milieu that blunts control (192). Together, these examples underscore that the clinical impact of HIV-associated viral interactions is often determined less by direct intracellular cooperation than by the durable immunological environment created by chronic HIV infection.

#### Beneficial interactions: GB virus C (GBV-C)

Not all HIV-associated viral interactions are pathogenic (Table 1). GBV-C is a human non-pathogenic lymphotropic flavivirus, the prevalence of which can reach up to 50% among people living with HIV-1, HCV, or HBV infection (76). Clinical studies have consistently shown that HIV-1/GBV-C coinfected individuals display lower plasma HIV-1 RNA levels, slower progression to AIDS, improved survival, and higher CD4^+^ T-cell counts compared to patients with HIV-1 only (193). Several mechanisms have been proposed to explain the protective effects of GBV-C. In HIV-1/GBV-C coinfected patients, circulating CD80^+^ pDCs are increased in number and activation state and correlate positively with higher CD4^+^ T-cell counts and negatively with viral load. *In vitro* studies of GBV-C/HIV-1 coinfection of PBMCs have shown that GBV-C infection induces increased expression and secretion of SDF-1, RANTES, MIP-1α, and MIP-1β chemokines. These chemokines can bind to both HIV-1 co-receptors, thus causing their downmodulation at the cell surface (194).

Notably, these examples show that the outcome of viral coinfection is dictated by timing, tissue context, and the local immune/interferon landscape. HIV-1 illustrates this especially well: through persistent replication, superinfection exclusion, receptor competition, genetic diversification, and immune remodeling, it can drive protection, persistence, or pathology. Although an RNA retrovirus, HIV-1 reshapes the ecological landscape for both RNA and DNA viruses across cellular, tissue, and systemic scales, including mucosal selection pressures and transmitted founder virus dynamics (195).

These examples demonstrate that tissue context, defined by cell-type composition, receptor availability, and intrinsic basal innate immune state, fundamentally shapes virus-virus interactions. Variations in cellular differentiation state, availability of replication factors, and heterogeneous expression of interferon-stimulated genes across mucosal and intestinal cell types create spatially distinct antiviral response dynamics that determine cell susceptibility to infection (196). Through local interferon signaling and paracrine effects on surrounding cells, tissues impose immunological and cellular constraints that govern whether viruses exclude one another, coexist, or facilitate replication.

## CLINICAL AND TRANSLATIONAL IMPLICATIONS

Beyond the immunological mechanisms discussed above, viral coinfection can translate these interactions into distinct clinical outcomes, influencing disease severity, immunopathology, and vaccine efficacy. Excessive immune activation during heterologous infections can amplify tissue damage. Under normal conditions, effector CD8^+^ T cells clear the infection and are then downregulated to prevent collateral damage due to excessive cytokine production by activated effector T cells. In contrast, dysregulated responses, such as those observed during DENV and influenza infections, can trigger a cytokine storm (197). In LCMV-immune mice infected with MCMV, memory T-cell infiltration was suppressed, viral loads increased, and immunopathology was aggravated (115). Dose also matters: in LCMV clone 13 infection, low doses elicit strong effector responses that clear virus; high doses cause exhaustion and persistence; intermediate doses may allow sufficient time for collateral tissue damage (97).

Importantly, innate immune-mediated effects likely operate alongside antibody-driven mechanisms *in vivo*, particularly during sequential infection with antigenically related viruses. In individuals previously exposed to DENV, cross-reactive but non-neutralizing antibodies can bind ZIKV and facilitate viral entry into Fc-receptor-expressing cells, a phenomenon known as antibody-dependent enhancement (198). Such antibody-mediated effects can counteract or override intrinsic antiviral states observed *in vitro*, contributing to increased viral replication, altered tissue tropism, and enhanced disease severity (64, 199).

### Vaccine-virus interactions

Another clinically important consequence of viral coinfections is the reduced efficacy of live attenuated virus vaccines (Table 1). In some cases, the coinfecting virus can suppress the replication of the live-attenuated vaccine virus, thereby limiting antigen availability and immune stimulation. Consistent with it, some published data suggest that recent SARS-CoV-2 variant infection, before vaccination with the live attenuated IAV vaccine strain, can suppress the replication of the vaccine strain in nasal epithelium (136). Furthermore, a similar effect was also observed for oral poliovirus vaccine and live attenuated rotavirus vaccines. Coinfection with non-polio enteroviruses prior to vaccination with live-attenuated virus vaccines can reduce the rate of seroconversion and antibody levels for poliovirus and rotavirus (83, 84). Importantly, such interference between live attenuated viruses also underlies standard vaccination practices, including the recommendation to separate administration of live attenuated vaccines (e.g., measles-mumps-rubella) by a minimum interval of 4 weeks to avoid mutual interference (200).

These clinical observations and vaccine schedule recommendations underscore how viral coinfection can shape immune responses and the effectiveness of prophylactic interventions in humans.

## CONCLUSION

Current evidence indicates that the outcome of viral co- and superinfection is determined primarily by three factors: (i) the temporal sequence of infections, particularly the duration of refractory periods, defined as transient post-infection intervals during which cells or tissues are resistant to secondary infection owing to sustained antiviral or ISG responses; (ii) the degree of cellular and tissue tropism overlap, including receptor and cofactor expression and compatibility of replication organelles; and (iii) the baseline immune activation state, encompassing constitutive interferon signaling, ISG expression, and adaptive immune memory from prior infections or vaccinations. At the cellular levels, SIE represents a virus-encoded regulatory mechanism that limits subsequent infection by modulating receptor availability, inhibiting membrane fusion, or restricting access to replication complexes. Through these processes, primary infection effectively restricts superinfection, reduces the opportunities for recombination, and maintains dominance of the initial viral population. When SIE is circumvented, via sequence variation, pseudotyping, or altered infection order, secondary infection can occur, allowing transient complementation, expanded host range, and increased genetic exchange.

**Fig 1:**
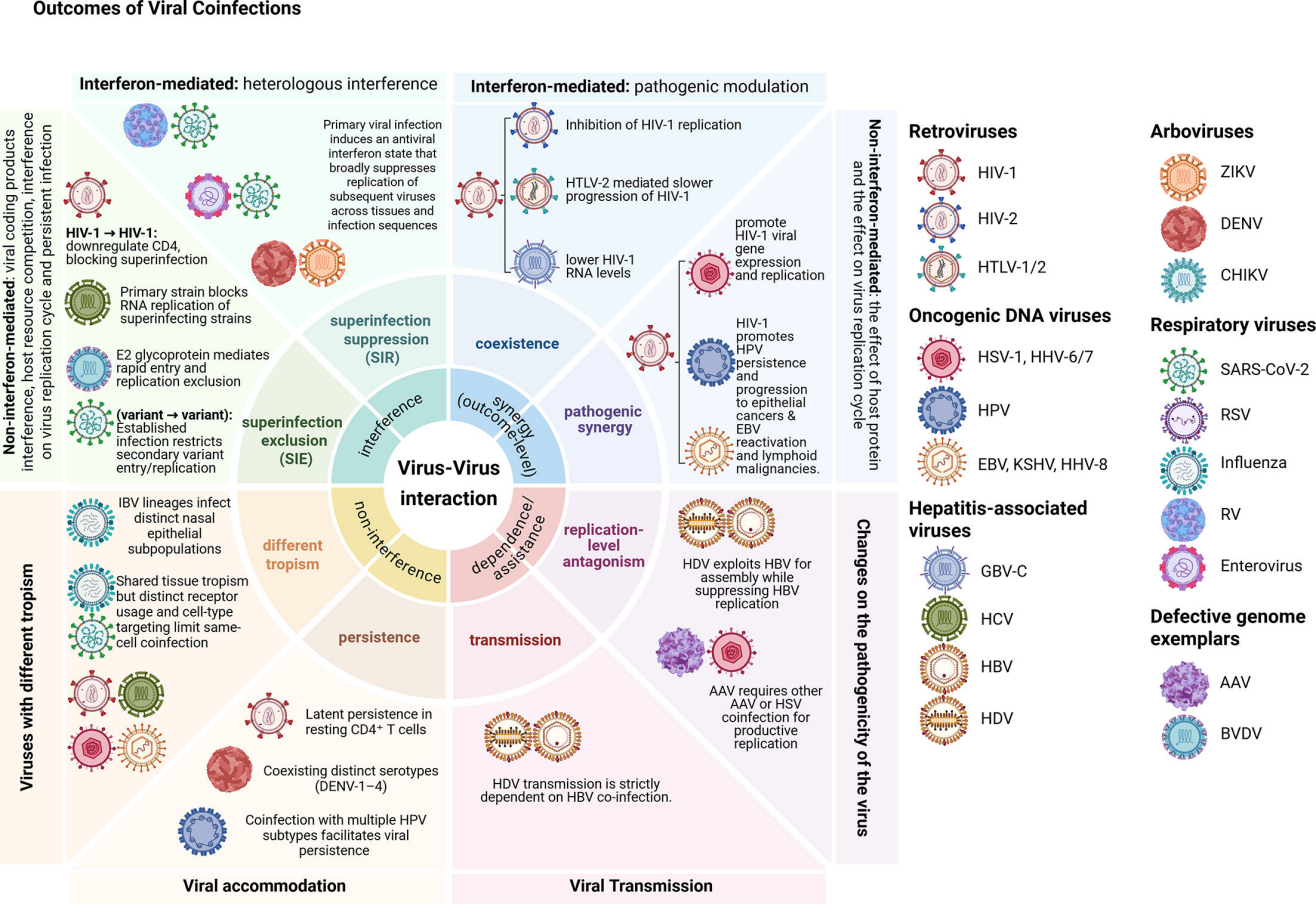
Outcomes of viral coinfections. Schematic overview of the major biological outcomes of virus-virus interactions during coinfection. Viral coinfections may result in interference, non-interference, dependence/assistance, or synergy, depending on viral tropism, replication dynamics, host antiviral responses, helper virus dependence, and effects on pathogenicity. Representative examples across retroviruses, oncogenic DNA viruses, hepatitis-associated viruses, arboviruses, respiratory viruses, and defective viral genomes are shown. Created in BioRender. Geijtenbeek, T. (2026) https://BioRender.com/nqt6d2d

At the host and population scales, these mechanisms manifest as viral interference, neutral coexistence, or synergistic infection, thereby influencing disease severity, transmission dynamics, lineage replacement, and vaccine-virus interactions (Fig. 1). Despite major advances, several key knowledge gaps remain. The frequent and spatial distribution of co- and superinfection events *in vivo* remains poorly quantified due to limited single-cell and spatially resolved data sets. Mechanistic understanding beyond interferon-mediated effects, such as competition for replication organelles, cytoskeletal remodeling, and autophagy-related restriction, is incomplete. In addition, integration of cellular-level SIE kinetics and refractory-period variability into epidemiological and evolutionary models remains limited, impeding accurate prediction of viral interference at the population level. Current research is heavily focused on a few RNA viruses (HIV, HCV, influenza, and SARS-CoV-2), leaving DNA viruses, arboviruses, and zoonotic reservoirs insufficiently studied, despite evidence that analogous exclusion mechanisms operate across taxa. Additional clinically relevant gaps include how co- and superinfections influence long-term sequelae, diagnostic interpretation of viral co-detection, prophylactic and vaccine strategies, and surveillance frameworks capable of distinguishing direct virus-virus interactions from broader changes in transmission ecology. Finally, existing models lack the spatiotemporal resolution required to observe dynamic viral interactions in intact tissues.

Progress in this field will depend on large and well-defined clinical cohort studies and quantitative, multiscale research that combines single-cell multi-omics, live-cell imaging, and computational modeling. These approaches will aid in defining how infection, timing, cellular tropism, and immune activation jointly shape viral coexistence. Such data-driven studies are essential to establish a predictive framework for virus-virus interactions and to identify opportunities for therapeutic exploitation of SIE to prevent viral recombination, limit pathogenesis, and improve vaccine design.
